# siRNA Mediate RNA Interference Concordant with Early On-Target Transient Transcriptional Interference

**DOI:** 10.3390/genes12081290

**Published:** 2021-08-23

**Authors:** Zhiming Fang, Zhongming Zhao, Valsamma Eapen, Raymond A. Clarke

**Affiliations:** 1 Ingham Institute, School of Psychiatry, University of NSW, Sydney, NSW 2170, Australia; z.fang@unswalumi.com (Z.F.); v.eapen@unsw.edu.au (V.E.); 2 Center for Precision Health, School of Biomedical Informatics and School of Public Health, The University of Texas Health Science Center at Houston, Houston, TX 77030, USA; zhongming.zhao@uth.tmc.edu

**Keywords:** transcriptional interference, siRNA, lncRNA, multiple synostosis syndrome type 4 (SYNS4), sets of interacting transcription units (SITRUS)

## Abstract

Exogenous siRNAs are commonly used to regulate endogenous gene expression levels for gene function analysis, genotype–phenotype association studies and for gene therapy. Exogenous siRNAs can target mRNAs within the cytosol as well as nascent RNA transcripts within the nucleus, thus complicating siRNA targeting specificity. To highlight challenges in achieving siRNA target specificity, we targeted an overlapping gene set that we found associated with a familial form of multiple synostosis syndrome type 4 (SYSN4). In the affected family, we found that a previously unknown non-coding gene *TOSPEAK/C8orf37AS1* was disrupted and the adjacent gene *GDF6* was downregulated. Moreover, a conserved long-range enhancer for *GDF6* was found located within *TOSPEAK* which in turn overlapped another gene which we named *SMALLTALK/C8orf37*. In fibroblast cell lines, *SMALLTALK* is transcribed at much higher levels in the opposite (convergent) direction to *TOSPEAK*. siRNA targeting of *SMALLTALK* resulted in post transcriptional gene silencing (PTGS/RNAi) of *SMALLTALK* that peaked at 72 h together with a rapid early increase in the level of both *TOSPEAK* and *GDF6* that peaked and waned after 24 h. These findings indicated the following sequence of events: Firstly, the siRNA designed to target *SMALLTALK* mRNA for RNAi in the cytosol had also caused an early and transient transcriptional interference of *SMALLTALK* in the nucleus; Secondly, the resulting interference of *SMALLTALK* transcription increased the transcription of *TOSPEAK*; Thirdly, the increased transcription of *TOSPEAK* increased the transcription of *GDF6*. These findings have implications for the design and application of RNA and DNA targeting technologies including siRNA and CRISPR. For example, we used siRNA targeting of *SMALLTALK* to successfully restore *GDF6* levels in the gene therapy of SYNS4 family fibroblasts in culture. To confidently apply gene targeting technologies, it is important to first determine the transcriptional interference effects of the targeting reagent and the targeted gene.

## 1. Introduction

Exogenous siRNAs are used routinely in many biotechnology and research applications to regulate endogenous gene expression levels. The most common is the use of siRNA for post transcriptional gene silencing (**PTGS**) via the knockdown of mRNA expression levels [1] which is also referred to as RNA interference (RNAi). Exogenous siRNA can also target nascent RNA transcripts in the nucleus with the potential to cause near permanent transcriptional silencing (**PTS**) or upregulation (**PTU**) of the associated gene through epigenetic modification of the DNA [2,3,4,5,6,7]. The mechanisms of siRNA mediated PTGS and PTS/PTU are well documented and involve siRNA targeting within the cytosol and the nucleus, respectively [1,2,3,4,5,6,7].

PTGS/RNAi represents the most widely documented siRNA approach for the transient reduction (knockdown) of target mRNA levels in the cytosol [1]. To achieve PTGS, an exogenous siRNA is designed to be complementary to a mature mRNA (exon derived) sequence. In contrast, for PTS/PTU, the siRNA are designed complementary to the immediate gene promoter or 3′ flanking region of the target gene, respectively [2,3,4,5,6,7]. During PTS/PTU, the exogenous siRNA enters the cytosol before relocating to the nucleus where it binds the nascent RNA transcript derived from the target gene causing epigenetic modification of the DNA of the target gene [5,8]. When successful, the resulting epigenetic modifications of the DNA in PTS/PTU can cause near permanent changes to the transcription of the target gene [8]. From these applications, it is obvious that siRNA target RNA transcripts within both the cytosol and the nucleus where the region of the RNA targeted determines the nature of the interference. We, therefore, questioned whether an siRNA designed for PTGS of a target mRNA in the cytosol could target and interfere with the transcription of the nascent RNA precursor of the target in the nucleus and whether it would be possible to differentiate between these two distinct on-target interference effects.

To differentiate between siRNA targeting effects on the mRNA and its nascent precursor, we determined to target one gene that overlaps another gene with the following specifications: Firstly, the transcription of the two overlapping genes must converge from opposite directions on the DNA so as to initiate RNA polymerase collision mediated transcriptional interference [9,10,11]. Secondly, the target gene must be expressed at higher levels than the gene it overlaps [11]. The rationale for this approach was that the more highly expressed of the two convergently transcribed genes has been shown to repress the transcription of the lower expressed gene [11] presumably through increased incidence of RNA polymerase collision events [10]. In this way, if the siRNA were to alter the transcription of the target gene (after binding its nascent RNA transcript) as reported elsewhere [11] this could in turn effect the transcription and expression of the overlapping gene [11] which could in turn be monitored using comparative rtPCR [11].

The Model: In this study, we discovered and were the first to characterize the long noncoding gene which we named *TOSPEAK*. We found that *TOSPEAK* was disrupted in a family with SYSN4 with multiple joint fusions, malformation of laryngeal cartilages and severe speech impairment. *TOSPEAK/C8orf37AS1* was found to overlap a nested long-range enhancer ECR5 that regulates expression of the adjacent bone morphogenetic protein gene *growth differentiation factor 6* (*GDF6*) [12]. This was of great interest as *GDF6* expression was previously found reduced in affected members of the SYSN4 family [13] thus raising the question as to whether the SYNS4 skeletal phenotype was a function of the *GDF6* or *TOSPEAK* genotype. Moreover, there was another interesting feature of *TOSPEAK* that warranted further investigation, namely that *TOSPEAK* physically overlaps another more highly expressed gene, which we named *SMALLTALK/C8orf37.*

To examine the expression of this overlapping gene set, we used siRNA to target *SMALLTALK* in primary fibroblast cell cultures. As expected, the siRNA targeting of *SMALLALK* resulted in a prolonged PTGS/RNAi mediated decrease in the mRNA level of *SMALLTALK*. In addition, we detected a transient increase in the mRNA levels of both *TOSPEAK* and *GDF6*. Together, our findings indicate a role for both *TOSPEAK* and *GDF6* in the joint, bone and cartilage malformations of the SYNS4 family [13]. Given that over 20% of human protein coding genes physically overlap [14] and that many others share promoters, and those like *GDF6* that have regulatory elements nested within adjacent genes, the findings of this study have important implications for genotype–phenotype correlation studies and gene targeting strategies and for the design of gene therapies including those that use exogenous siRNA.

## 2. Materials and Methods

RNA isolation and cDNA synthesis: Total RNA was extracted from tissues (fresh skin biopsies and primary fibroblast cell lines derived there from, and from fresh white blood cells) using Trizol following the manufacture’s protocol (ThermoFisher Scientific Australia, Sydney, Australia). RNA was treated with DNase I (NEB Biolabs, Ipswich, Australia), ethanol precipitated, resuspended in DEPC-treated water and quality tested using spectrophotometry (A260/280 ratio was ~1.7) and gel electrophoresis. Then, 1 ug of total RNA extracted was reverse transcribed using 250 ng of random hexamers (Promega Pty Ltd., Sydney, Australia) in a standard 20 μL reaction including 4 μL of first strand buffer (Invitrogen Pty Ltd.), 2 μL of 0.1M DDT, 1 μL of 10 mM dNTP, 1 μL RNase inhibitor (2500 U) and 1 μL of reverse transcriptase (10,000 U) (Invitrogen Pty Ltd.). After annealing of the hexamers for 10 min at 72 °C, cDNA synthesis was performed for 42 °C for 90 min followed by an enzyme inactivation step at 70 °C for 15 min. All cDNA products were diluted in a ratio of 1:10 and stored at −20 °C before use.

TOSPEAK transcription start site and termination site: 1 ug of total RNA was reversed transcribed using 1 μL of reverse transcriptase (10,000 U) (ThermoFisher Scientific Australia, Sydney, Australia), where each reaction was primed with 1 μL of 12 mM 5′-CDS primer A (5′-(T) 25VN-3′) (ThermoFisher Scientific Australia, Sydney, Australia) and 1 mL of 12 mM SMART II A oligo (5′-AAGCAGTGGTATCAACGCAGAGTACGCGGG-3′) (Clontech Pty Ltd.).

After annealing of hexamers for 10 min at 70 °C, cDNA synthesis was performed using Superscript II (Invitrogen Pty Ltd.) at 42 °C for 90 min followed by enzyme inactivation at 72 °C for 7 min with addition of 100 μL of Tricine-EDTA buffer. The 5′RACE clones were amplified with a reverse primer from *TOSPEAK* exon 6 (Table 1) using 10× Universal Primer A mix as per manufacturers’ protocol (ThermoFisher Scientific Australia, Sydney, Australia)). PCR was performed using the following conditions: 5 cycles at 94 °C for 30 s and 72 °C for 2 min, 5 cycles at 94 °C for 30 s, 70 °C for 30 s and 72 °C for 2 min, and 30 cycles at 94 °C for 30 s, 68 °C for 30 s and 72 °C for 2 min. 3′-RACE libraries were generated from RNA with Superscript III (Invitrogen Pty Ltd.: Cat No. 18080-093) using primers and protocols described in the SMART RACE User Manual (Becton Dickinson, Sydney, Australia). The 3′RACE clones were amplified with a forward primer from *TOSPEAK* exon 9 (Table 1). RACE PCR products were excised from gels and cloned into the pGEMT vector (Promega, Sydney, Australia) and sequenced using Big Dye chemistries (Australian Genome Research Facility, Brisbane, Australia).

RT-PCR characterisation of *TOSPEAK* transcripts: RT-PCR reactions contained 5 μL of the diluted cDNA template, 2.5 μL of 10× PCR buffer, 0.2 μL of 25 mM dNTPs, 1 μL of each of the forward and reverse primer stocks (10 mM) (Table 1), 1.5 μL of 25 mM MgCl_2_ and 0.25 μL of AmpliTaq Gold polymerase (Applied Biosystems, Sydney, Australia) made up to 25 μL with ddH_2_O and amplified using an initial denaturation at 94 °C for 10 min followed by 40 cycles at 94 °C for 30 s,58 °C for 30 s and 72 °C for 40 s and a final extension of 72 °C for 15 min.

Antibody Screen: An affinity purified polyclonal antibody was raised in rabbit against a synthetic peptide *CESFLRKSVALPGEVIKSLLA* (Monash University Melbourne, Australia) that we generated based on part of a putative ORF from the most abundant human *TOSPEAK* transcript (Genebank accession number GU295154). This antibody was used to screen human lymphocytes using Western analysis (Z.F. & R.A.C.) and paraffin embedded human heart tissue using immunohistochemistry (St George Hospital Clinical Pathology Laboratory, Sydney, Australia). Note: This antibody was affinity purified against the original synthetic peptide antigen; however, no positive control tissue sample was available to test the functional validity of this antibody in western analysis or immunohistochemistry and this antibody displayed negative staining in all tissues interrogated (results not shown).

Comparative genomic analyses: Nucleotide sequences from a ~900 kb region of the genome spanning the *TOSPEAK* gene locus, were extracted from the Ensembl and NCBI GenBank databases (http://www.ensembl.org/ (accessed on 21 August 2009)), version 41.36c; http://www.ncbi.nlm.nih.gov/, Build 37.1 (accessed on 21 August 2009), for human, chimpanzee, dog, mouse, and opossum and analysed for any evolutionary conservation using VISTA (http://genome.lbl.gov/vista/ (accessed on 21 August 2009)) [15] with the human as the reference sequence.

Cell lines: Fresh skin biopsies were used to generate primary fibroblast cell lines cultured at 37 °C in DMEM media with 10% fetal calf serum (FCS). A commercial skin fibroblast cell line was used as independent control NC1 (NHDF-c adult normal human dermal fibroblast cell line PromoCell-Bio Connect C-12302). To harvest cells for Western analysis, washed cells were disrupted by adding 100 μL of lysis buffer (300 mM NaCl, 1 mM EDTA, 30 mM Tris/HCl, 1 μL proteinase inhibitor) and stored on ice for 30 min. For rtPCR cells were centrifuged at 1000 rpm for 5 min and resuspended and washed in PBS before RNA isolation.

siRNA mediated transient transcriptional interference (TTI) protocol: At sub-confluence, fibroblast cultures were washed with cold PBS and harvested using 0.25% trypsin in PBS at 37 °C for 1–5 min. Trypsin was then deactivated by suspension of cells in DMEM containing 10% FCS. Cells were centrifuged at 1000 rpm for 7 min and resuspended in DMEM without FCS before replating in preparation for treatment with siRNAs. Cells were plated into 6 well culture plates at 10^5^ cells/well in 2 mL and incubated for 17–24 h prior to treatment with siRNA.

Working stocks of STEALTH siRNAs (Table 2—Life Sciences Corp) were prepared at a 1/200 dilution (2 μM) in nuclease free H_2_O and stored at −80°C. Then, 2.5 μL of siRNA working stock was added to 95 μL serum-free media. At the same time 5 μL Lipofectamine 2000 (Invitrogen Pty Ltd.) was added to 95 μL serum-free media and gently mixed. The siRNA mix and Lipofectamine mix were then combined and incubated for 20 min, then transferred to ‘treatment’ wells containing fibroblasts (1 mL FCS free media + 2.5 μL siRNA (5 nM) + 5 μL Lipofectamine Reagent 2000) and incubated at 37 °C for 6 h before the addition of 1 mL of DMEM containing 20% FCS. After 18 h cell culture media was replaced with fresh DMEM containing 10% FCS (referred to as time zero). Cells were then incubated for 24 h (Time 1), 48 h (Time 2) or 72 h (Time 3) before harvesting for RNA extraction and real time rtPCR analysis—cell culture media was then removed and adherent cells washed twice with PBS at room temperature before adding 350 μL of cell lysis buffer (2.4 mL Buffer RLT and 20 μL of β-Mercaptoethanol) to each well (as per RNeasy Mini Kit (50) #74104, Qiagen, Melbourne Australia). All siRNA experiments were performed in triplicate.

*TOSPEAK* was not amenable to siRNA knockdown in our hands (results not shown). Note: This may have been due to the newly evolved *TOSPEAK* gene being very poorly conserved in sequence and structure between species with only 2 permanently transcribed exons (exons 1 and 9, respectively) that are very short and highly enriched for GC dinucleotides and repetitive elements (see Results below). However, siRNA targeting of the coding gene *SMALLTALK* was successful (using siRNA-S1, -S2 and -S3—see Table 2) and achieved peak silencing of *SMALLTALK* within ~72 h.

Comparative RT-PCR: First-Strand cDNA Synthesis was performed using the SuperScript™ III First-Strand synthesis qRT-PCR Kit (ThermoFisher Scientific Australia, Sydney, Australia) according to manufacturers’ instructions: 10 μL of 2X RT Reaction Mix, 2 μL RT Enzyme Mix and 50 pg of RNA were made up to 20 μL with DEPC-treated water and incubated at 25 °C for 10 min and again at 42 °C for 50 min. Reactions were terminated at 85 °C for 5 min, then chilled on ice for 5 min followed by a short spin in the microfuge. Then, 1 μL (2 U) of E. coli RNase H was added and incubated at 37 °C for 20 min. A qPCR master mix was prepared with all common components. Volumes for a single 25 μL reaction were 12.5 μL of Platinum^®^ SYBR^®^ Green qPCR SuperMix-UDG (ThermoFisher Scientific Australia, Sydney, Australia), 1 μL each of 10 μM primer stocks specific for gene of interest (Table 1), 2.5 μL of cDNA and DEPC-treated water to 25 μL. Reactions were incubated at 50 °C for 2 min and an initial denaturation step of 94 °C for 2 min. qPCR was performed for 40 cycles: denature at 94 °C for 15 s, anneal at 55 °C for 10 s, extension at 72 °C for 20 s. Comparative rtPCR profiles were independently normalised against expression of *GAPDH* and *18sRNA* to remove the non-biological variation. Each of the experimental triplicates were evaluated in triplicate (technical triplicates) and expressed as the mean. Patient rtPCR data was expressed as the mean of 2 patients.

## 3. Results

### 3.1. Genomic Analysis of the SMALLTALK-TOSPEAK-GDF6 Gene Locus

**Figure 1:**
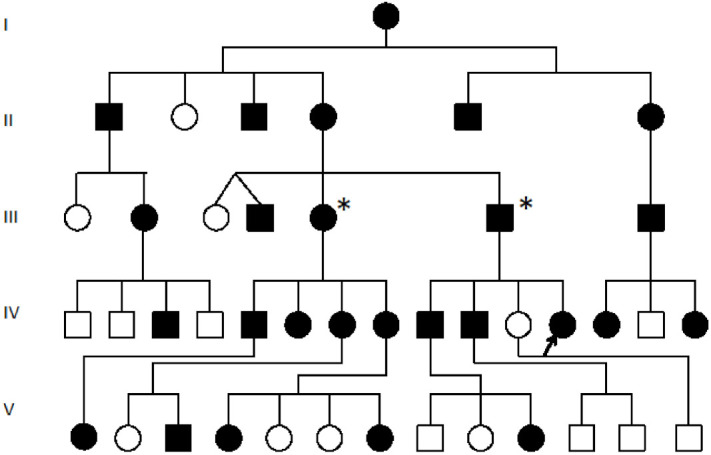
Pedigree of the SYNS4 family with congenital carpal tarsal coalition and progressive vertebral and ossicle joint ossification: All affected family members (filled symbols) presented with a degree of vertebral fusion and disruption of the *TOSPEAK* gene [17]. Nearly 50% of affected family members tested presented with bilateral fusion of carpal and tarsal joints [17]. The severity of phonological speech impairment was varied and more severe in affected males in association with ossification and malformation of laryngeal cartilages and ligaments [18]. Square symbols (Males), Circle symbols (Females), Filled symbols (Affected). Blank circles (Unaffected), Proband (Arrowed for fibroblast cell line testing), * (Fresh Blood testing for GDF6 expression).

**Figure 2:**
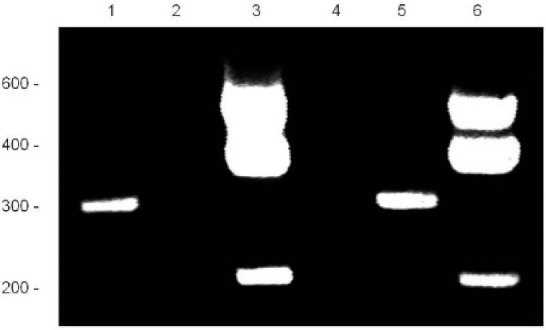
RACE Analysis of *TOSPEAK*: RACE analysis of mRNA was used to identify the boundaries of the *TOSPEAK* gene. 5′ RACE (Lanes 1 & 5) and 3′ RACE (Lanes 3 & 6) identified the start site and termination sequence of *TOSPEAK*, respectively. RACE products were PCR amplified using Elongase enzyme (Lanes 1 & 3) and Titanium enzyme (Lanes 5 & 6) using *TOSPEAK*-2 forward and reverse primer sets, respectively (Table 1).

**Figure 3:**
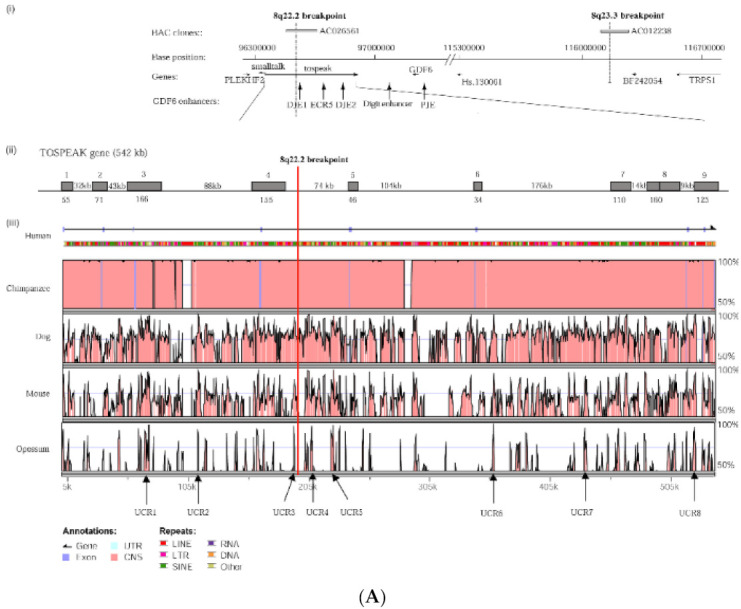

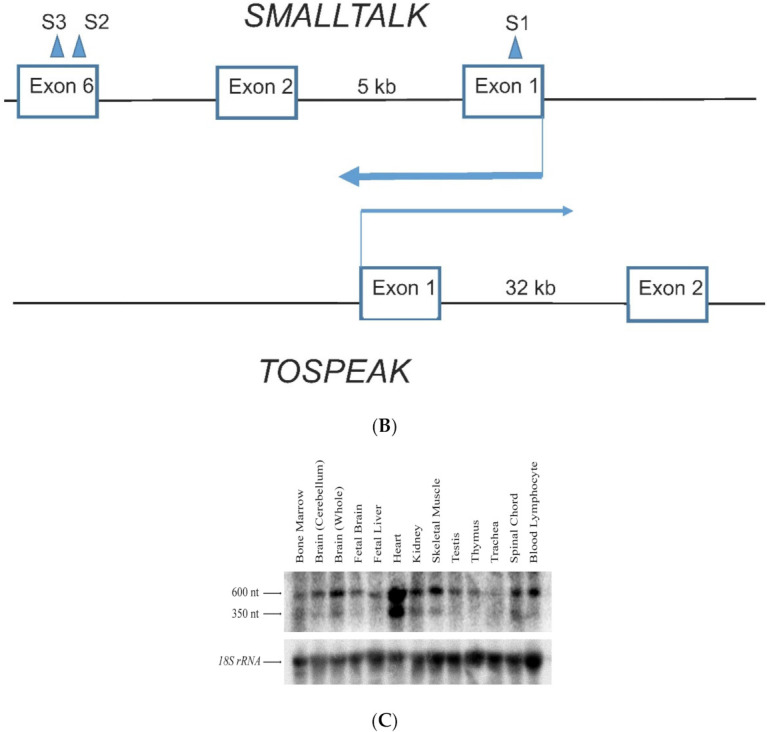
*TOSPEAK* gene characterisation: (**A**) Comparative genomic analysis of the *TOSPEAK* locus (**i**) Schematic view of the genomic region spanning inv(8) (q22.2q23.3) breakpoints in the SYNS4 family with congenital carpal tarsal coalition and progressive postnatal ossification and fusion of vertebral and ear joints. Genes (horizontal arrows), *GDF6* enhancers (vertical arrows). (**ii**) *TOSPEAK*/*C8orf37-AS1* gene structure with 8q22.2 breakpoint in the 4th intron. (**iii**) VISTA plot spanning *TOSPEAK* gene (exons marked blue) for multiple vertebrate species where strict selection criteria applied for highly conserved regions (HCRs ≥ 200 bp ungapped alignment with >90% identity). The human gene annotation was obtained from the Ensembl database and the repeat information was obtained from Repeat Masker (6 September 2009)). (**B**) Overlap between the *TOSPEAK* and *SMALLTALK/C8orf37* genes. (**C**) Northern Analysis of *TOSPEAK* in human tissues.

In an earlier study, a breakpoint on chromosome 8q between the *SMALLTALK* gene and the *GDF6* bone morphogenetic protein gene was identified segregating with vertebral fusion and malformation of laryngeal cartilages in a family with SYNS4 (Figure 1) [13,16,17]. Located near the breakpoint were three short expressed sequence tags (ESTs): BU570390, AI832412 and AV713874. In the present study, we investigated the origin of these ESTs using RACE, RT-PCR and nucleotide sequence analysis (Figure 2). We established that the three ESTs derived from a hitherto unknown gene which we named *TOSPEAK/C8orf37-AS1. TOSPEAK* was found to overlap the 5′ end of *SMALLTALK/C8orf37* such that *TOSPEAK* was transcribed in the opposite convergent direction to *SMALLTALK* (Figure 3) [16,17].

**Figure 4:**
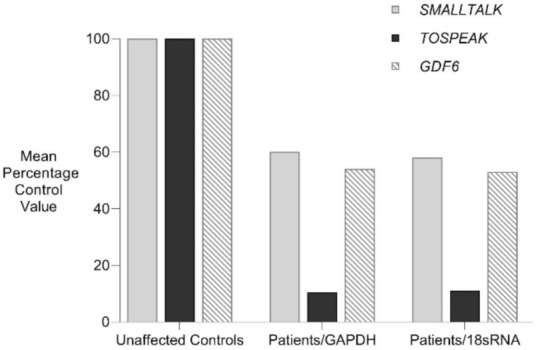
Familial analysis of *TOSPEAK*, *GDF6* and *SMALLTALK* expression. Comparative rtPCR expression analyses of *TOSPEAK*, *GDF6* and *SMALLTALK* in fresh white blood cells from two affected family members were compared with five age and gender matched unaffected controls independently normalised against the expression of control genes *GAPDH* and *18sRNA* and expressed as the mean percentage of normal control levels.

*TOSPEAK* spans 542 kb of genomic DNA between *SMALLTALK* and *GDF6* (Figure 3). *TOSPEAK* has nine short exons of which all but the first and last exons (exons 1 and 9, respectively) are alternatively spliced-out, leading to numerous short, low-abundance transcripts of between 180 and 600 nucleotides in length. Northern blot analysis indicated ubiquitous low-level expression of two major overlapping *TOSPEAK* transcripts in human adult and neonatal tissues (Figure 3C). All *TOSPEAK* transcripts contained an excessive number of stop codons and none of the transcripts encoded for any recognisable protein structure. Furthermore, the polyclonal antibody that we raised in rabbit against a synthetic peptide corresponding to part of the most abundant of two short open reading frames of *TOSPEAK* did not detect a protein in Western analysis of lymphocytes and showed no positive staining in the immunohistochemistry of heart tissue sections (results not shown). As such *TOSPEAK* exhibited all the features of a long non-coding transcription unit (lncRNA gene). Further analysis found that *TOSPEAK* harbours the highly conserved ECR5 long-range enhancer for *GDF6* [12]. ECR5 is located in one of *TOSPEAK’s* introns (Figure 3A). As such *TOSPEAK* is transcribed across the ECR5 enhancer which regulates *GDF6* transcription in the developing pharyngeal arches [12], which in human give rise to the laryngeal structures malformed in the speech affected family [18]. Furthermore, *GDF6, TOSPEAK* and *SMALLTALK* expression levels were reduced in fresh white blood cells of two affected family members compared with five unaffected control individuals (Figure 4) [13]. 

### 3.2. siRNA Mediate RNAi and Transient Transcriptional Interference

**Figure 5:**
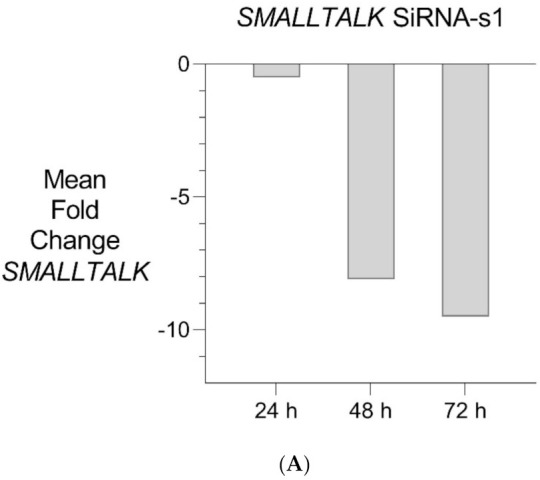

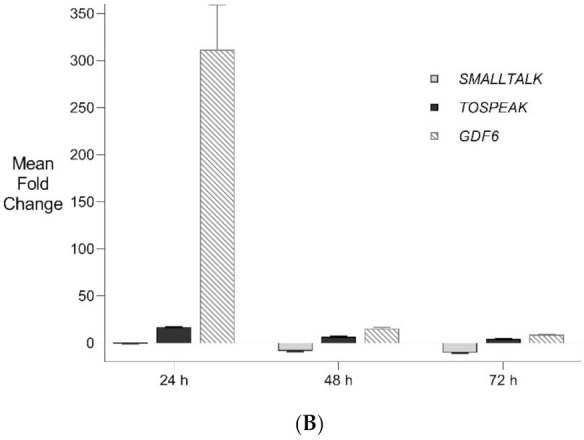
Transient Transcriptional Interference. (**A**) siRNA mediated knockdown of *SMALLTALK*: Comparative rtPCR expression analysis of *SMALLTALK* in the normal human fibroblast cell line (NC1) over 72 h following exposure to siRNA-S1 targeting *SMALLTALK,* expressed as the mean fold change relative to the mean for untreated control levels. (**B**) siRNA mediated transient transcriptional interference of *SMALLTALK*: Comparative rtPCR expression analysis of *SMALLTALK, TOSPEAK* and *GDF6* in the normal human fibroblast cell line (NC1) over 72 h following exposure to siRNA-S1 targeting *SMALLTALK* expressed as the mean fold change relative to untreated control levels.

**Figure 6:**
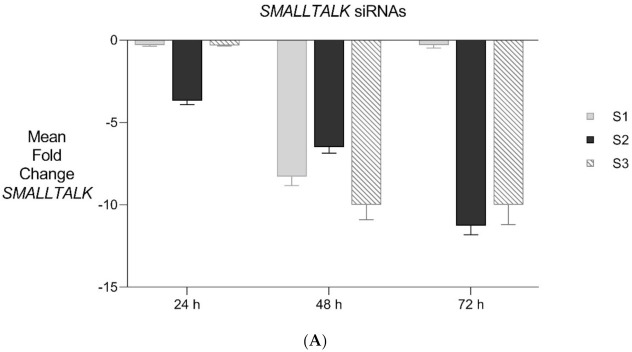

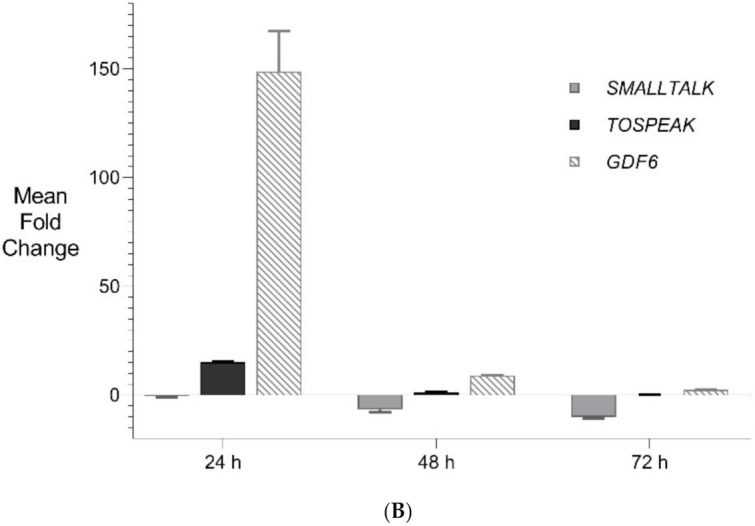
Expanded Application of Transient Transcriptional Interference. (**A**) Expanded siRNA Mediated Knockdown of *SMALLTALK*: Comparative rtPCR expression analysis of *SMALLTALK* in the normal human fibroblast cell line (NC1) over 72 h following separate exposure to siRNA-S1, siRNA-S2 and siRNA-S3 targeting *SMALLTALK*, respectively, expressed as the mean fold change of untreated control levels. (**B**) siRNA Mediated Transient Transcriptional Interference Assays: Comparative rtPCR expression analysis of *TOSPEAK*, *GDF6* and *SMALLTALK* in normal human fibroblasts over 72 h following exposure to siRNA-S2 targeting *SMALLTALK* expressed as the mean fold change relative to the mean for untreated control levels.

PCR gene expression analysis in fibroblast cultures indicated that *SMALLTALK* was expressed at much higher levels compared with either *TOSPEAK* or *GDF6* (results not shown). As *SMALLTALK* appeared to be transcribed at higher levels convergent with *TOSPEAK* we targeted *SMALLTALK* using a series of siRNA (S1–S3). These siRNAs were designed complementary to the mRNA of *SMALLTALK* (Table 2) to target that region of *SMALLTALK* well clear of the overlap region with *TOSPEAK* (Figure 5). As expected, siRNA-S1 mediated a reduction in *SMALLTALK* levels that peaked at 72 h (Figure 5A). Surprisingly, the siRNA-S1 silencing of *SMALLTALK* was associated with a clear increase in the level of *GDF6* at 72 h but not *TOSPEAK* (Figure 5B). We tested for temporal changes in the expression of *GDF6* and *TOSPEAK* at earlier time points during the assay, again using siRNA-S1 to target *SMALLTALK* (Figure 5B). At 24 h both *GDF6* and *TOSPEAK* expression had increased dramatically long before the maximum silencing of *SMALLTALK* at 72 h (Figure 5A,B). We repeated this experiment using two different siRNA (siRNA-S2 and siRNA-S3) to target *SMALLTALK*, (Table 2, Figure 6A). Both siRNA-S2 & S3 also induced a rapid, concordant and proportional induction of both *TOSPEAK* and *GDF6* expression within 24 h comparable to the result for siRNA-S1 (Figure 6B). Moreover, the induced levels of both *TOSPEAK* and *GDF6* dissipated rapidly after 24 h prior to the peak reduction in *SMALLTALK* at 72 h (Figure 6B).

### 3.3. siRNA Reverse Downregulation of GDF6 in SYNS4 Family Fibroblasts

**Figure 7:**
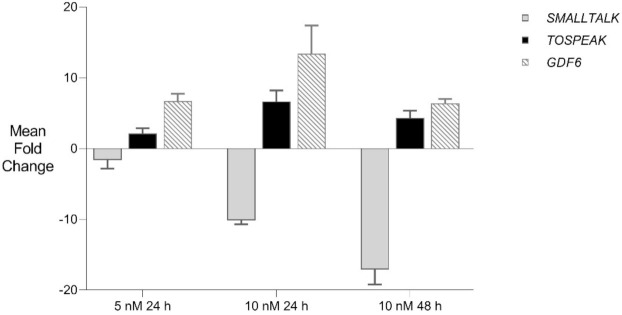
*GDF6* Gene Therapy: siRNA mediated transcriptional interference and comparative rtPCR expression analysis of *TOSPEAK*, *GDF6* and *SMALLTALK* in fibroblast cell line derived from a severely SYSN4 affected family member following exposure to *SMALLTALK* siRNA-S2 (5 nM) and (10 nM) expressed as the mean fold change relative to the mean for untreated control levels.

We then used siRNA-S2 to target *SMALLTALK* in primary fibroblast culture derived from a severely speech affected member of the SYNS4 family in which a copy of the *TOSPEAK* gene had been disrupted. This resulted in the concordant and proportional induction of both *TOSPEAK* and *GDF6* levels which peaked within ~24 h. Despite this, concordant induction of *TOSPEAK* and *GDF6* in family fibroblasts came off a much lower base compared to that in normal fibroblasts—which indicated that the familial breakpoint within *TOSPEAK* had caused a reduction in the level of both *TOSPEAK* and *GDF6* transcription (Figure 1 and Figure 7). To test if we could modulate the level of *GDF6* induction in fibroblasts from the affected family we doubled the assay concentration of siRNA-S2 from 5 nM to 10 nM. The increased concentration of siRNA -S2 resulted in a greater induction of both *TOSPEAK* and *GDF6* by ~24 h which were again both concordant and proportional and dissipated rapidly after 24 h (Figure 7). In all assays, the induction of *TOSPEAK* and *GDF6* was transient and dissipated rapidly from peak levels at ~24 h whereas *SMALLTALK* levels continued to decrease for the duration of the 72 h assay (Figure 5, Figure 6 and Figure 7).

## 4. Discussion

PTGS/RNAi reduces the level of target mRNAs in the cytosol. To achieve PTGS, the siRNA is designed to be complementary to a target site within the target mRNA, despite the fact that an identical target site also exists within the immature and/or intermediate nascent RNA transcript precursor of that mRNA within the nucleus. Due to this potential for dual targeting of both the mRNA and its precursor nascent transcript by a single siRNA, the following questions arise: Firstly, to what extent if any does an siRNA designed for PTGS (target and reduce the level of a specific mRNA) also target the identical site within the nascent RNA transcript precursor of that mRNA? Secondly, to what extent if any does this affect the transcription of that gene? Thirdly, given that any such reduction in transcription will also reduce the level of the target mRNA is it possible to distinguish any such effect on transcription from the PTGS/RNAi effect on the mRNA?

In this study, three distinct siRNA were used to target *SMALLTALK*. For each of the three siRNA, there were three distinct responses. The first response was the knockdown of *SMALLTALK* levels which peaked near 72 h in typical fashion to that expected for PTGS/RNAi in the cytosol. The second and third responses were the early transient increase of *TOSPEAK* and *GDF6* levels, respectively, both of which peaked simultaneously at ~24 h before declining thereafter. The second and third responses were therefore independent of the PTGS of *SMALLTALK* which continued unabated for another 48 h. As such, the increase in *TOSPEAK* is best explained by its convergent transcriptional overlap with the *SMALLTALK* gene. Convergent transcription between overlapping genes results in RNA polymerase collision events [10,11] that can cause discordant transcriptional interference [9,10,11]. Other examples of transcriptional interference between convergently transcribed overlapping genes include the *DLX1*, *DLX5* and *DLX6* genes which all experience transcriptional interference from overlapping non-coding antisense genes [9]. However, for such a mechanism to be in play in our experiments would require the prior interference of *SMALLTALK* transcription by the siRNA-S1-3. Support for this scenario came from the discordant increase in the level of *TOSPEAK* as *SMALLTALK* levels decreased during the first 24 h of the assay. Moreover, a similar sequence of events to this was observed with respect to the convergent transcription of the overlapping *LRRTM3* and *CTNNA3* genes associated with autism [11]. In that study, five different siRNA were used to target the more highly expressed *CTNNA3* gene which in all five cases resulted in discordant transient interference (increase) of *LRRTM3* transcription [11]. Together, these results are consistent with the siRNA mediated interference (reduction) of *SMALLTALK* transcription causing reduced transcriptional repression (and increase) of *TOSPEAK* [5,8].

**Figure 8:**
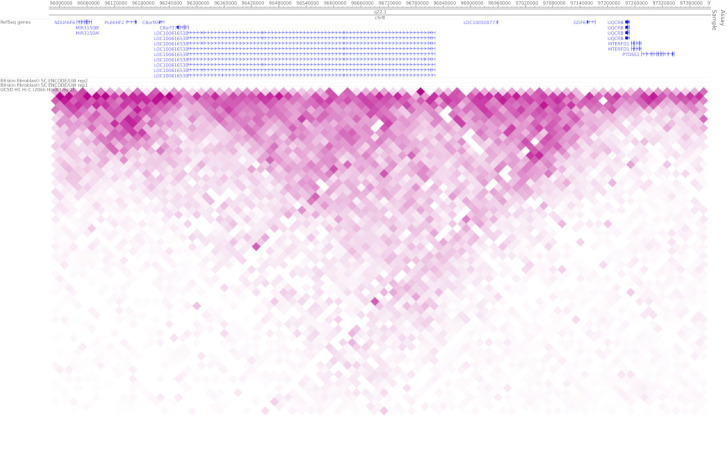
Genome-wide 4C and Hi-C interaction across the *SMALLTALK/TOSEPAK/GDF6* locus provided by the Encode & NIH Roadmap projects available at http://promoter.bx.psu.edu/hi-c/view.php (accessed on 10 August 2021).

Further support for the transcriptional interference of *TOSPEAK* by *SMALLTALK* comes from the third response which was the concordant, proportional and transient increase in *GDF6*. *TOSPEAK* is transcribed across the highly conserved ECR5 long-range enhancer of *GDF6* and both *TOSPEAK* and *GDF6* levels increased in response to the siRNA-S targeting of *SMALLTALK*. Within the 1st 24 h of the siRNA-S assay there was a synchronous, proportional and transient induction of both *TOSPEAK* and *GDF6* levels followed by a concordant and proportional decline of both *TOSPEAK* and *GDF6* levels within the following 24 h well before the peak reduction in *SMALLTALK* levels at 72 h (Figure 7 and Figure 8). This strongly suggested that *TOSPEAK* transcription, not the *TOSPEAK* transcript, is a positive regulator of *GDF6* transcription. This interpretation of the results is also consistent with the phenotypic findings from the speech affected SYSN4 family where the breakpoint in the *TOSPEAK* gene, which blocked transcription across ECR5, was associated with reductions in both *TOSPEAK* and *GDF6* expression levels (Figure 4) and the aberrant ossification of the very same joints, ligaments and cartilages regulated by the *GDF6* enhancer [13,19,20]. 

In summary, these findings suggest that:

1. siRNA-S targeting of *SMALLTALK* interferes with *SMALLTALK* transcription.
2. *SMALLTALK* transcription converges on & represses *TOSPEAK* transcription.
3. *TOSPEAK* transcription across *GDF6* enhancer enhances *GDF6* transcription.

A number of possible mechanisms have been suggested for the modulation of highly conserved enhancers such as ECR5 by the transcription of non-coding genes like *TOSPEAK* [9]. Included is the possibility that *TOSPEAK* transcription across ECR5 may enhance *GDF6* transcription through the secondment of the *GDF6* enhancer into active transcription factories, thereby facilitating *GDF6* promoter–enhancer coupling and increased transcription of *GDF6* [9]. This scenario may involve modulation of chromatin structure around the enhancer [9]. A similar scenario has been demonstrated for transcription-enhancer overlaps elsewhere [21]. Furthermore, genome-wide 4C and Hi-C interaction data mark the locus spanning *SMALLTALK*, *TOSPEAK* and *GDF6* as a functionally interactive chromatin domain (Figure 8) consistent with the three overlapping genes functioning as a set of overlapping interacting transcription units [10,11,21].

It has been shown elsewhere that not all overlapping genes or gene sets are implicated in this form of transcriptional interference [11]. Furthermore, it is uncertain to what degree this phenomenon is limited by cell or tissue specificity [9,11]. Furthermore, additional studies are required to understand the mechanism by which siRNA designed for PTGS/RNAi interfere with the transcription of the parent gene which in this case was *SMALLTALK* [2,3,4,5,6,7].

## 5. Conclusions

We discovered and were the first to characterize the long noncoding gene which we named *TOSPEAK* and its physical overlap with both the *SMALLTALK* gene and the ECR5 long-range enhancer for *GDF6.* Furthermore, we established that *TOSPEAK* was disrupted in the SYNS4 family with reduction of both *TOSPEAK* and *GDF6* levels. We used this overlapping set of genes to demonstrate siRNA mediated on-target transcriptional interference and its relevance for gene function and genotype–phenotype association analysis and gene therapy as evidenced by the restoration of *GDF6* levels in cells from the SYNS4 family.

The limitations of this study included the use of one specific cell type in culture conditions different to those in vitro. Further studies are required to understand the mechanism by which siRNA designed for RNAi cause transient transcriptional interference of their target nascent transcript [2,3,4,5,6,7]. Uncertainty also remains regarding the mechanism by which *TOSPEAK* could induce the transcription of *GDF6*. Highly conserved lncRNA transcripts often have important regulatory functions [9]; however, the *TOSPEAK* transcript is not conserved between species. Moreover, *TOSPEAK* gives rise to numerous very short transcripts with no translated protein structure and a very high incidence of stop signals making the mature transcript (s) of *TOSPEAK* a most unlikely regulator of *GDF6*. Notwithstanding, the nascent transcript of *TOSPEAK* does include the sequence of the highly conserved ECR5 enhancer of *GDF6* and as such could feasibly have a trans role in enhancing *GDF6* transcription [9]. Notwithstanding the most plausible interpretation of the results are that *TOSPEAK* transcription, not the *TOSPEAK* transcript, positively regulates *GDF6* transcription.

## Figures and Tables

**Table 1:** Primer Table.

Primer	Sequence (5′→3′)
*GDF6*–forward	CCTGTTGCTTGTTTGGTTCA
*GDF6*–reverse	GCTGTCCATTTCCTCTTTGC
*SMALLTALK*–forward	AGTAGCGACCGGAACCAAG
*SMALLTALK*–reverse	TTGTCCAAGTTGGGCTCTTC
*TOSPEAK* (exon 6)–reverse	GCAGGTCCTTGAATCCCTCATGGCCAT
*TOSPEAK* (exon 9)–forward	TATGCTTCACAGGTGTTTCT
*TOSPEAK1* (exon 8)–forward	TGGCAGTTCCATCATTTGAA
*TOSPEAK1* (exon 9)–reverse	AGGAGAAACACCTGTGAAGCA
*TOSPEAK2* (exon 9)–forward	AGCTCTCCTGGCATACTCTGA
*TOSPEAK2* (exon 9)–reverse	CCCAGATGGGATGAGACATA
18S rRNA–forward	GTAACCCGTTGAACCCCATT
18S rRNA–reverse	CCATCCAATCGGTAGTAGCG
*GAPDH*–forward	CCACCCATGGCAAATTCCATGGCA
*GAPDH*–reverse	TCTAGACGGCAGGTCAGGTCCACC

**Table 2:** siRNAs.

siRNA (Target)	Sequence (5′→3′)
siRNA-S1 (*SMALLTALK* exon 1)–sense	CAAGCCAAGGCGAAAGAGACGCUCA
siRNA-S1 (SMALLTALK exon 1)–antisense	UGAGCGUCUCUUUCGCCUUGGCUUG
siRNA-S2 (*SMALLTALK* last exon)–sense	CCAGUGUAGCUGGAGAACUAUUGAA
siRNA-S2 (*SMALLTALK* last exon)–antisense	UUCAAUAGUUCUCCAGCUACACUGG
siRNA-S3 (*SMALLTALK* last exon)–sense	UCGCUGGGUUUGUGGUAAACAUUAA
siRNA-S3 (*SMALLTALK* last exon)–antisense	UUAAUGUUUACCACAAACCCAGCGA
siRNA-T1 (*TOSPEAK intron 1*)–sense	AUCACUGCCAGUUUCUACACCUCUG
siRNA-T1 (*TOSPEAK intron 1*)–antisense	CAGAGGUGUAGAAACUGGCAGUGTU
Stealth control–sense	CAAGAACAGCGAGAAGCAGCCGUCA
Stealth control–antisense	UGACGGCUGCUUCUCGCUGUUCUUG

## References

[1] Cheng J.C., Moore T.B., Sakamoto K.M. (2003). RNA interference and human disease. Mol. Genet. Metab..

[2] Schwartz J.C., Younger S.T., Nguyen N.-B., Hardy D.B., Monia B.P., Corey D.R., Janowski B.A. (2008). Antisense transcripts are targets for activating small RNAs. Nat. Struct. Mol. Biol..

[3] Elbashir S.M., Harborth J., Lendeckel W., Yalcin A., Weber K., Tuschl T. (2001). Duplexes of 21-nucleotide RNAs mediate RNA interference in cultured mammalian cells. Nature.

[4] Green V.A., Weinberg M.S. (2011). Small RNA-induced transcriptional gene regulation in mammals mechanisms, therapeutic applications, and scope within the genome. Prog. Mol. Biol. Transl. Sci..

[5] Matsui M., Prakash T.P., Corey D.R. (2013). Transcriptional Silencing by Single-Stranded RNAs Targeting a Noncoding RNA That Overlaps a Gene Promoter. ACS Chem. Biol..

[6] Yue X., Schwartz J.C., Chu Y., Younger S.T., Gagnon K.T., Elbashir S., Janowski B.A., Corey D.R. (2010). Transcriptional regulation by small RNAs at sequences downstream from 3’ gene termini. Nat. Chem. Biol..

[7] Moazed D. (2009). Small RNAs in transcriptional gene silencing and genome defence. Nature.

[8] Castel S.E., Martienssen R.A. (2013). RNA interference in the nucleus: Roles for small RNAs in transcription, epigenetics and beyond. Nat. Rev. Genet..

[9] Fatica A., Bozzoni I. (2014). Long non-coding RNAs: New players in cell differentiation and development. Nat. Rev. Genet..

[10] Prescott E.M., Proudfoot N.J. (2002). Transcriptional collision between convergent genes in budding yeast. Proc. Natl. Acad. Sci. USA.

[11] Fang Z.M., Eapen V., Clarke R.A. (2017). CTNNA3 discordant regulation of nested LRRTM3, implications for autism spectrum disorder and Tourette syndrome. Meta Gene.

[12] Reed N.P., Mortlock D.P. (2010). Identification of a distant cis-regulatory element controlling pharyngeal arch-specific expression of zebrafish gdf6a/radar. Dev. Dyn..

[13] Clarke R.A., Schirra H.J., Catto J.W., Lavin M.F., Gardiner R.A. (2010). Markers for Detection of Prostate Cancer. Cancers.

[14] Chen C.H., Pan C.Y., Lin W.C. (2019). Overlapping protein-coding genes in human genome and their coincidental expression in tissues. Sci. Rep..

[15] Frazer K.A., Pachter L., Poliakov A., Rubin E.M., Dubchak I. (2004). VISTA: Computational tools for comparative genomics. Nucleic Acids Res..

[16] Clarke R.A., Singh S., McKenzie H., Kearsley J.H., Yip M.Y. (1995). Familial Klippel-Feil syndrome and paracentric inversion inv(8)(q22.2q23.3). Am. J. Hum. Genet..

[17] Tassabehji M., Fang Z.M., Hilton E.N., McGaughran J., Zhao Z., de Bock C.E., Howard E., Malass M., Donnai D., Diwan A. (2008). Mutations in GDF6 are associated with vertebral segmentation defects in Klippel-Feil syndrome. Hum. Mutat..

[18] Clarke R.A., Davis J., Tonkin J. (1994). Klippel-Feil syndrome associated with malformed larynx. Case report. Ann. Otol. Rhinol. Laryngol..

[19] Mortlock D.P., Guenther C., Kingsley D.M. (2003). A general approach for identifying distant regulatory elements applied to the Gdf6 gene. Genome. Res..

[20] Settle S.H., Rountree R.B., Sinha A., Thacker A., Higgins K., Kingsley D.M. (2003). Multiple joint and skeletal patterning defects caused by single and double mutations in the mouse Gdf6 and Gdf5 genes. Dev. Biol..

[21] Miles J., Mitchell J.A., Chakalova L., Goyenechea B., Osborne C.S., O’Neill L., Tanimoto K., Engel J.D., Fraser P. (2007). Intergenic transcription, cell-cycle and the developmentally regulated epigenetic profile of the human beta-globin locus. PLoS ONE.

